# Investigation of Potential Antioxidant, Thrombolytic and Neuropharmacological Activities of *Homalomena aromatica* Leaves Using Experimental and In Silico Approaches

**DOI:** 10.3390/molecules26040975

**Published:** 2021-02-12

**Authors:** Md. Sekendar Ali, Syed Al Jawad Sayem, Habibullah, Yixian Quah, Eon-Bee Lee, Biruk Tesfaye Birhanu, Kyoungho Suk, Seung-Chun Park

**Affiliations:** 1 Department of Biomedical Science, School of Medicine, Brain Science and Engineering Institute, Kyungpook National University, Daegu 41944, Korea; alipharm2000@gmail.com (M.S.A.); ksuk@knu.ac.kr (K.S.); 2 Department of Pharmacology, School of Medicine, Brain Science and Engineering Institute, Kyungpook National University, Daegu 41944, Korea; 3 Department of Pharmacy, International Islamic University Chittagong, Kumira, Chittagong 4318, Bangladesh; aljawadsayem@gmail.com (S.A.J.S.); habibmahirbd@gmail.com (H.); 4 Laboratory of Veterinary Pharmacokinetics and Pharmacodynamics, College of Veterinary Medicine, Kyungpook National University, Daegu 41566, Korea; im.yixianquah@gmail.com (Y.Q.); eonbee@gmail.com (E.-B.L.); btbtes@gmail.com (B.T.B.)

**Keywords:** *H. aromatica*, antioxidant, thrombolytic, anxiolytic, antidepressant, essential oil, molecular docking, ADME profiling

## Abstract

The leaves of *Homalomena aromatica* are traditionally used in Bangladesh for the treatment of different chronic ailments. The purpose of this study was to explore in vitro antioxidant, thrombolytic activities, and in vivo neuropharmacological effects of methanolic extract of *Homalomena aromatica* (MEHA) leaves. Antioxidant activity of MEHA was assessed by a DPPH free radical scavenging assay and total phenolics content, total flavonoids content were also measured. The thrombolytic activity was determined by percentage of clot lysis and neuropharmacological activities by hole board, tail suspension, forced swimming and elevated plus maze tests. The results showed that the IC_50_ value of the extract against DPPH was 199.51 μg/mL. Quantitative analysis displayed higher contents of phenolics and flavonoids (147.71 mg gallic acid equivalent/g & 66.65 mg quercetin equivalent/g dried extract, respectively). The extract also showed a significant clot lysis (33.31%) activity. In case of anxiolytic activity, the elevate plus maze (EPM) test demonstrated an increase in time spent in open arms, and in case of hole board test, the number of head dipping was also significantly increased (*p* < 0.05). All the test compared with control (1% Tween in water) and standard (diazepam 1 mg/kg), significant dose (200 & 400 mg/kg) dependent anxiolytic activity was found. In antidepressant activity, there was a significant decrease in period of immobility in both test models (tail suspension and forced swimming) (*p* < 0.05). Moreover, 13 compounds were identified as bioactive, showed good binding affinities to xanthine oxidoreductase, tissue plasminogen activator receptor, potassium channel receptor, human serotonin receptor targets in molecular docking experiments. Furthermore, ADME/T analysis revealed their drug-likeness, likely pharmacological actions and non-toxic upon consumption. Taken together, our finding support the traditional medicinal use of this plant, which may provide a potential source for future drug discovery.

## 1. Introduction

Thrombosis is a blood clotting disorder and one of the leading causes of death in developed countries [1]. Besides modern treatment strategies, traditional and herbal drugs could be the potential alternatives to treat thrombosis for their safety profiles.

Neuropsychiatric disorders such as depression are a common but serious mood illness resulting in severe symptoms such as sadness, feelings of guilt, loss of interest affecting sleeping, eating and other daily activities. If untreated, depression along with anxiety may aggravate the symptoms, worsen the complications, and ultimately lead to suicidal ideation risks [2]. The real etiology of depression and anxiety are still not clearly known, however they may arise due to disruption of the antioxidant defence system which eventually causes redox imbalance or oxidative stress [3]. The excessive production of oxidative reactive species (ROS) in the brain creates a cellular discrepancy condition that causes cognitive deteriorations and neurological mechanism damage [4]. Antioxidant therapies may be used to ameliorate the progressive tissue damage, preventing imbalance of ROS production in the nervous system. The beneficial effects in mild depressive conditions of using currently available antidepressants is still in doubt and moreover, they also produce many unwanted side effects [5]. On the other hand, taking only antioxidants may not produce the expected therapeutic effect. Therefore, pharmacotherapy for a longer period of time, for example co-administration of antioxidants along with psychiatric agents is suggested to treat such critical health diseases. In this circumstance, the investigation of possible bioactive leads from medicinal plants covering multiple pharmacological targets is a main aim of worldwide research, because medicinal plants some of the most abundant sources of pharmacological active compounds for the development of a new drugs. Nowadays, in vitro and in vivo model in silico methods are also widely used to scrutinize the biological activities of phytochemicals with better safety, economic and drug-likeliness properties [6].

*Homalomena aromatica* (Spreng.) Schott is a perennial medicinal herb belonging to the Araceae family that occurs in the Chittagong hills region in Bangladesh. It has many traditional usages, for instances, management of coughs, colds, stomach problems, skin diseases and jaundice [7]. It is also used by the tribal habitants in the hilly areas of the eastern regions of India (Sikkim, Arunachal) and Bhutan as an anti-inflammatory, antidepressant, analgesic, sedative, antispasmodic, antiseptic and pain killing agent [7]. There are a lot of scientific studies which have showed that *H. aromatica* has diverse pharmacological activities like antibacterial [8], ulcer protective [9] and hepatoprotective effects [10]. GC-MS analysis has revealed that specific compounds are present in *H. aromatica* leaves, most of them in the form of essential oils [11]. Besides essential oils, it also contains alkaloids, saponins, cardioactive glycosides, polyphenolics, fixed oils, oleic acid, undecylenic acid, epiandrosterone, palmitic acid and linoleic acid [12]. Among them, we have selected 13 compounds for in silico studies to verify their target receptor binding affinity to prove the basis of their potential antioxidant, thrombolytic, antidepressant and anxiolytic activities. Previous studies have shown the best activity of certain compounds of this plant such as T-cadinol, which is an antioxidant and thrombolytic compound [13], 4-terpineol that has anxiolytic activity [14], and α-cadinene that exerts antidepressant effects [15].

Nonetheless, while plant has many traditional usages, as yet, according to our knowledge, there are no exclusive scientific investigations which have been carried out to search for antioxidant, thrombolytic and neuropharmacological activities of this plant along using in silico approaches. Therefore, our current study has been designed to systematically explore the antioxidant, thrombolytic, antidepressant and anxiolytic activities along with in silico methods to reveal the potential target insights of the documented bioactive compounds for potentially finding lead constituents from *H. aromatica* leaves.

## 2. Results and Discussion

Historically plants are the major source of invaluable therapeutic agents for the treatment of different diseases [16]. Natural products generate a wide range of interest to discover new drugs, even in this modern age, because plants are magnificent sources of biomolecules providing new pathways to treat many chronic ailments [17]. Nowadays, available drugs on the market for the treatment of thrombosis include antiplatelet agents (aspirin, thienopyridine) and anticoagulants (heparin, warfarin, hirudin, human activated protein C) that act by preventing blood clotting. There are also a number of clinically available antidepressant and anxiolytic drugs, apparently acting via different mechanisms, including dopaminergic, noradrenergic or serotonergic pathways. However, these drugs have limitations including insufficient efficacy over a longer period of time and unexpected side effects [18]. Therefore, in recent years many researchers have explored alternative source of drugs that seemingly show less or no side effects. Many natural herbal plants, for example, *Chaenomeles sinensis*, *Spatholobus suberectus*, *Salvia miltiorrhiza*, *Garcinia nervosa*, *Piper longum*, *Agrimonia pilosa*, *Licania pittieri* have been investigated for thrombolytic effects [19]; and *Crocus sativus*, *Passiflora incarnata*, *Piper methysticum* have been tested for their anxiolytic and antidepressant activities [20,21]. The presence of antioxidants, alkaloids and many other phenolic and flavonoid compounds are reported to have anti-nociceptive, anti-inflammatory, antimicrobial, anticancer and thrombolytic activities [22].

In order to develop herbal drugs for thrombosis, anxiety and depression the use of animal models are inevitable. Therefore, validated experimental protocols using experimental animals are a relevant contributor for preclinical and clinical inference. In this study, we have used in vitro and in vivo along with in silico approaches to investigate the possible antioxidant, antidepressant, anxiolytic and thrombolytic activities of MEHA.

### 2.1. In Vitro Antioxidant Activity

#### DPPH Free Radical Scavenging Assay

**Figure 1:**
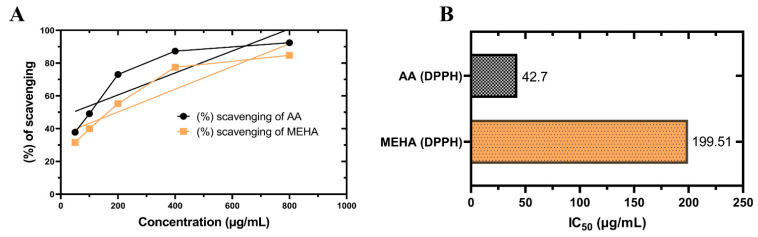
DPPH free radical scavenging activity of MEHA compared with the reference standard ascorbic acid (AA). (**A**) Percentage of DPPH free radical scavenging activity by different concentrations of the MEHA and reference standard AA. (**B**) IC_50_ values for DPPH free radical scavenging activity of MEHA and AA. Values are expressed as mean ± SEM of three independent experiments. MEHA refers to methanol extract of *Homalomena aromatica* leaves. DPPH: 1,1-diphenyl-2-picrylhydrazyl radical.

Imbalances between the production of ROS and intracellular antioxidants provoke the formation of excessive reactive species leading to the process of atherothrombosis via release of pro-thrombotic and inflammatory molecules [23,24]. Similarly, excessive production of ROS and meager antioxidant defenses in the brain make it highly vulnerable to the pathophysiology of different diseases. Evidence suggests that oxidative stress is connected to anxiety-related behavior by destabilizing neurotransmission and brain activities [25]. An oxidant can prevent or delay the oxidation of a substrate by hydrogen transfer, single oxygen transfer, chelating peroxidase metals, inactivating lipoxygenase and thus can prevent any abnormal harms into the cells [26]. Results for the DPPH free radical scavenging activity of MEHA are shown in Figure 1. The extract showed an efficient scavenging effect in the DPPH assay. The half inhibition concentration (IC_50_) for free radicals achieved by MEHA is 199.51 μg/mL which is statistically significant compared to that (IC_50_ = 42.70 μg/mL) of ascorbic acid (reference compound).

The result proves that the antioxidant capability of MEHA can be regarded as a potential antioxidant remedy for thrombosis, anxiety and depression. Regular supplementation of antioxidants may provide the desired outcome due to the presence of polyphenols providing natural defenses against these diseases.

### 2.2. Total Phenolics and Flavonoids Content

Polyphenols are plant secondary metabolites that provide potential health benefits by dietary intake acting as potential antioxidants [27], which may boost the brain energy by scavenging ROS and inhibit the inflammation signaling system ameliorating depression and anxiety [28]. Flavonoids are compounds which can reduce neuroinflammation, potentiate the GABA_A_-Cl^−^ channel complex, modulate the monoaminergic neurotransmitter and increase dopamine, serotonin, and monoamines levels in the central nervous system [29]. Polyphenols have also gained researchers’ attention as potential therapeutic agents which act by combating oxidative stress to protect from cardiovascular diseases [30]. These compounds prevent the development of atherosclerosis and protect the endothelium and reduce the oxidation of LDL and cholesterol [31]. We have analyzed and measured the amount of polyphenols (Table 1) measured as total phenolics content (147.716 ± 5.07 mg GAE/g) and total flavonoids content (66.65 ± 6.208 mg QE/g dried extract). These results provide the information that MEHA could be the potential therapeutic target for the treatment of neuropsychiatric disorders and thrombosis.

### 2.3. Thrombolytic Activity

**Figure 2:**
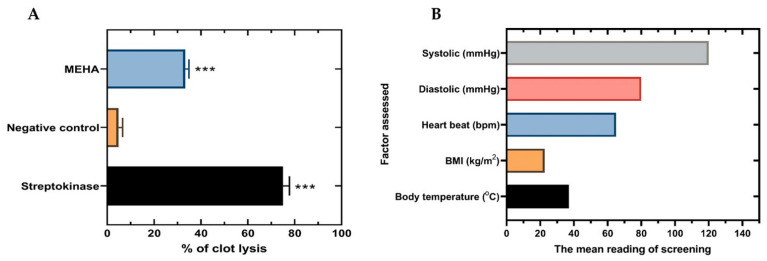
(**A**) The clot lysis activity of MEHA, streptokinase (SK) and distilled water (negative control). (**B**) The healthy volunteer status presented. Results are expressed as mean ± SEM of three independent experiments. *** *p* < 0.001, significantly different from control; ANOVA followed by Dunnett’s test. MEHA: Methanol extract of *Homalomena aromatica* leaves.

Formation of blood clots is one of the chief causes of blood circulation problems. Thrombi or emboli can be deposited in the blood vessels and can obstruct blood flow in that location depriving tissues from normal blood flow and oxygen. Ultimately, this can result in damage, destruction or even death of the tissues in that area. Thrombolysis is the process by which blood clots can be destroyed by administering thrombolytic drugs including recombinant tissue plasminogen activator, which reinforce the normal destruction of blood clots by the body’s enzymes, however, this may cause an increased risk of bleeding so it is usually used only for specific situations (such as massive pulmonary embolism and severe strokes). Plants are natural sources of many remedies including ones for the treatment of thrombosis. There are many research works that have reported the discovery of plants with thrombolytic activity and the use of such plants as a source of medicines that may lead to the prevention and treatment of strokes and coronary disease [32,33]. It has been demonstrated that phenolics and flavonoids obtained from plant sources might have significant thrombolytic activity potential [34]. The in vitro thrombolytic activity assays revealed that HEMA showed 33.31% clot lysis effect (*p* < 0.001) which was significant compared to negative control (4.84%), whereas the standard streptokinase used as positive control showed 75% clot lysis (Figure 2).

### 2.4. Anxiolytic Activity

**Figure 3:**
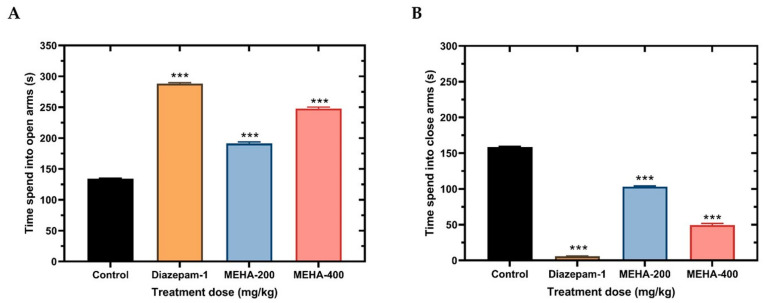
Anxiolytic activity of MEHA on elevated plus maze test in mice. Time spend in open arm (**A**), time spends in closed arms (**B**). Results are expressed in mean ± SEM. *** *p* < 0.001, significantly different from control; ANOVA followed by Dunnett’s test (*n* = 3, per group). MEHA: methanol extract of *Homalomena aromatica* (200 mg/kg and 400 mg/kg); Reference drug diazepam 1 mg/kg.

Amongst many significant animal experiments, elevated plus maze (EPM) is one of the most important behavioral assays for the assessment of anxiolytic-like effects. The sensitivity of this test to the effect of anti-anxiety and anxiogenic drugs acting to the GABA benzodiazepine complex is very high [35]. The EPM apparatus has two opposite open and two closed arms, where regular mice normally spend much of their allowed time in the closed arms. This type of behavior reflects a distaste for open arms created by the anxiety caused by open spaces. Drug-like anxiolytics that enhance the exploration of open arms are believed to have anxiolytic activity. Administration of extract significantly increased the time spent in open arms compared to the control (Figure 3). As shown in the Figure 3A, MEHA at the dose of 200 mg/kg and 400 mg/kg significantly enhanced the time spent (*p* < 0.001) in the open arms (191.33 ± 2.43 and 248 ± 2.08 s, respectively) indicating anxiolytic effects. Understandably, the time spent in the closed arms of extract treated mice were less (Figure 3B).

**Figure 4:**
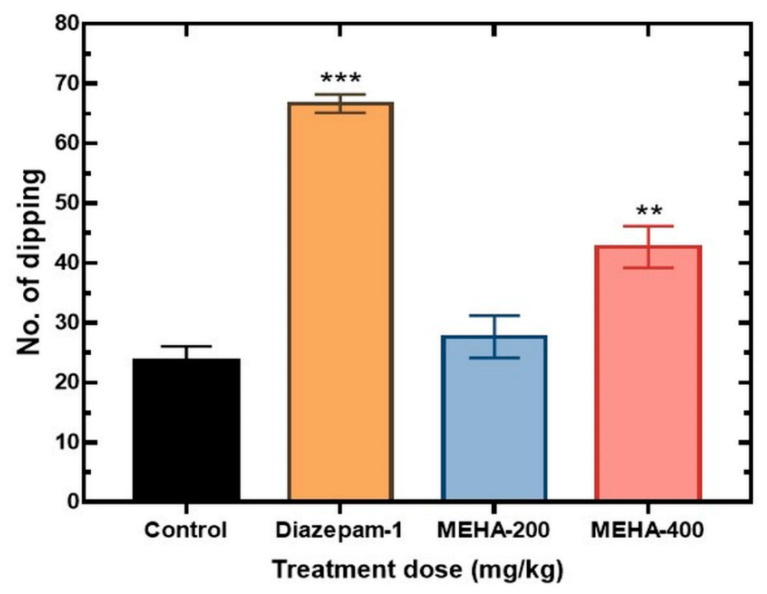
Anxiolytic activity of MEHA on hole board test in mice. Results are expressed in mean ± SEM. ** *p* < 0.01 and *** *p* < 0.001, significantly different from control; ANOVA followed by Dunnett’s test (*n* = 3, per group). MEHA: methanol extract of *Homalomena aromatica* leaves (200 mg/kg and 400 mg/kg); Reference drug diazepam 1 mg/kg.

Likewise, the hole board experiment used to show the anxiolytic effect of the extract. After treatment, the head dipping number was increased efficiently in case of diazepam as well as extract administered animals compared to the control. The MEHA at both dose levels showed an increase in the number of head dipping events whereas 400 mg/kg produced a significant upsurge (42.67 ± 3.48) as compared to the control animals (*p* < 0.01) (Figure 4).

### 2.5. Antidepressant Activity

In our exploration, besides anxiolytic activity, MEHA showed antidepressant activity which was evaluated through forced swim and tail suspension tests. These two tests are very convenient and the most widely used validated animal models for the evaluation of substances with potential antidepressant-like activities as well as to identify the pathological mechanism(s) of depression [36,37]. 

**Figure 5:**
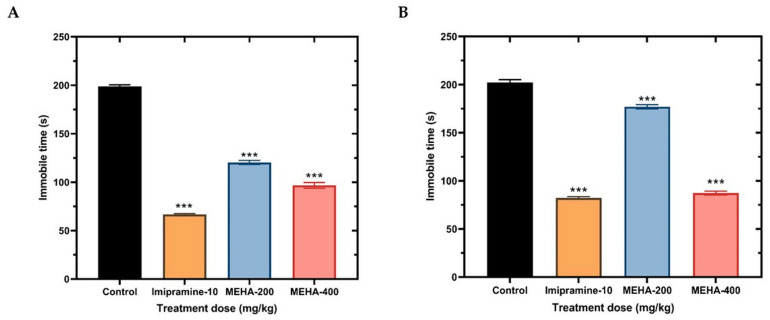
Antidepressant activity of MEHA on tail suspension (**A**) and forced swimming (**B**) tests in mice. Results are expressed in mean ± SEM. *** p < 0.001, significantly different from control; ANOVA followed by Dunnett’s test (*n* = 3, per group). MEHA: methanol extract of *Homalomena aromatica* leaves; MEHA-200, methanol extract of *H*. *aromatica* 200 mg/kg; MEHA-400, methanol extract of *H*. *aromatica* 400 mg/kg; Reference drug imipramine hydrochloride (10 mg/kg).

A recent investigation suggest that stressful conditions activate the hypothalamic-pituitary-adrenal axis which in turn stimulates the periventricular neurons to release corticotrophin-releasing factors [38]. As it is well known depression symptoms are revealed due to overactivation of the hypothalamic-pituitary-adrenal axis, causing abnormal regulation of corticotrophin-releasing factors resulting in the suppression of the adrenocorticotropic hormone response and elevated cerebrospinal fluid levels [39]. Moreover, several neurotransmitters including serotonin, noradrenalin, dopamine, and GABA are involved in the pathophysiology of depressive illness. Thus, it is also believed that depression is due to the deficiency of one or another of these neurotransmitters [40]. Successful antidepressants act by suppressing stress-induced hypothalamic-pituitary-adrenal axis activation and restoring the normal flow of neurotransmitters [41]. In forced swim experiments, when mice are forced to swim in a confined condition, they become immobile after an initial period of struggling. This inevitable stressful condition is evaluated as the parameter to measure the activity of antidepressant agents. In this test (Figure 5A), animals treated with two doses of MEHA (200 and 400 mg/kg, p.o) showed decreases in their immobility times, which was significant (120.33 ± 2.03 s and 96.67 ± 2.96 s, respectively) when compared with control (198.67 ± 1.76 s). Animals treated with imipramine hydrochloride (10 mg/kg), as expected, showed a significant decrease in their immobility time (66.67 ± 0.89 s). Similarly, the tail suspension assay is another primary screening test for detecting antidepressant activity of a substance. This test induces a state of despair in animals similar to that of forced swim test. In the tail suspension test (Figure 5B), animals were treated with 200 and 400 mg/kg doses of MEHA showed decreases in their immobility times (177.00 ± 2.16 s and 87.33 ± 1.96 s, respectively) which was significant as compared to control (202.33 ± 2.896 s), while animals treated with imipramine hydrochloride (10 mg/kg), also showed a significant decrease in their immobility time (82.33 ± 1.29 s). This evidence suggest that MEHA has the ability to suppress depression and anxiety in mice.

### 2.6. In Silico Studies

Contacts between ligand and protein are fundamental to all organic processes. By means of these interactions, living entities maintain all types of regulatory and metabolic systems that together organize their lifecycle processes. To predict and understand all off these biological processes, computer-aided analyses: molecular docking, homological modeling, molecular dynamics and simulations are widely used scientific tools because these methods are economic and time saving. The target molecules can be also utilized as bioactive substances for modification and controlling the bio-mechanism in an in silico medium. To make our findings more relevant, we conducted an in silico molecular docking investigation targeting xanthine oxidoreductase (PDB: 2CKJ) to determine antioxidant properties, tissue plasminogen activator receptor (PDB: 1A5H) for thrombolytic activity, potassium channel receptor (PDB: 4UUJ) for anxiolytic properties, and human serotonin receptor (PDB: 5I6X) for antidepressant activity. In this analysis, four widely used standard agents—ascorbic acid, streptokinase, diazepam and imipramine hydrochloride—were selected to compare the docking scores with the compounds obtained from essential oils from the leaves of *H. aromatica*.

**Figure 6:**
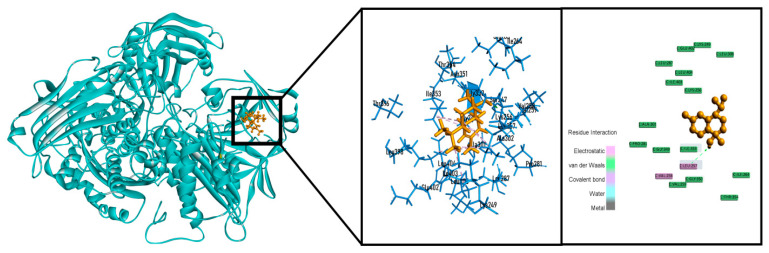
Best docked poses and 2D interactions of T-cadinol with xanthine oxidoreductase (PDB: 2CKJ) for antioxidant activity.

In the case of the computer-aided antioxidant docking study, the outcomes of the docking investigation against xanthine oxidoreductase for antioxidant activity are shown in Table 2. 4-Terpineol (−6.457 kcal/mol) and T-cadinol (−6.882 kcal/mol) showed better docking scores than ascorbic acid (−6.407 kcal/mol). Contrariwise, bullatantriol (−6.260 kcal/mol) and oplodiol (−6.182 kcal/mol) also demonstrated good binding scores close to ascorbic acid. T-Cadinol showed the best docking score among all compounds in our antioxidant assays along with standard drugs by interacting with one hydrogen bond (LEU257) and six hydrophobic bonds (LEU257, ALA301, ILE353, LEU257, ILE403 and LEU404) showed in Figure 6. The compound named α-cadinene did not bind with xanthine oxidoreductase.

**Figure 7:**
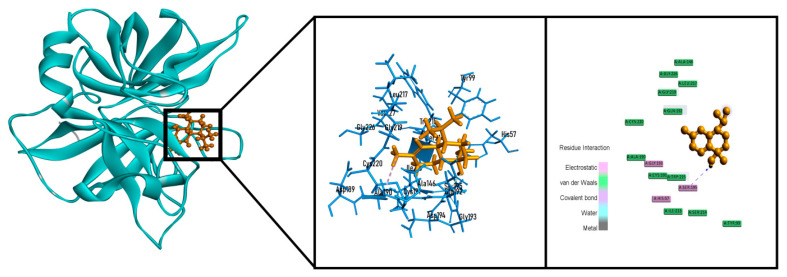
Best docked poses and 2D interactions of T-cadinol tissue plasminogen activator receptor (PDB: 1A5H) for thrombolytic activity.

In thrombolytic docking assay, 4-terpineol (−5.254 kcal/mol), α-cadinene (−5.720 kcal/mol), spatulenol (−6.190 kcal/mol) and T-cadinol (−6.300 kcal/mol) showed better biding affinity than streptokinase (−5.102 kcal/mol) against tissue plasminogen activator receptor (Table 3). Among them T-cadinol exhibited the best result by interacting with two hydrogen bonds (SER195 and GLN192) and one hydrophobic bond (CYS220) (Figure 7).

**Figure 8:**
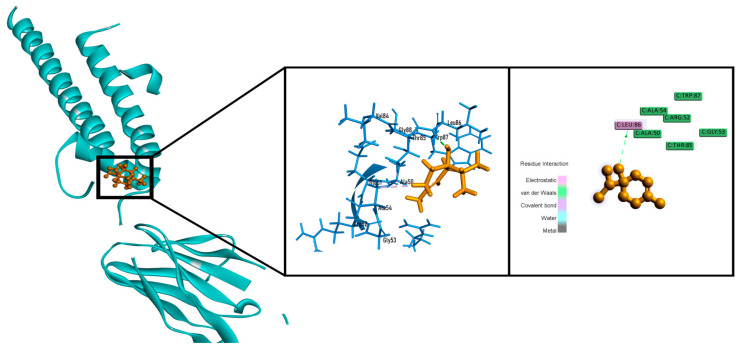
Best docked poses and 2D interactions of 4-terpineol with potassium channel receptor (PDB: 4UUJ) for anxiolytic activity.

In our anxiolytic docking investigation 1, 2-dimethylbenzene (−3.658 kcal/mol), 4-terpineol (−5.143 kcal/mol), α-cadinene (−3.442 kcal/mol), bullatantriol (−4.098 kcal/mol), cryptone (−3.512 kcal/mol) and α-linalool oxide (−3.691 kcal/mol) demonstrated better binding attraction than diazepam (−3.140 kcal/mol) against potassium channel receptor (Table 4). Whereas, 4-terpineol showed best score by interacting with one hydrogen bond (leu86) and one hydrophobic bond (ALA54) (Figure 8).

**Figure 9:**
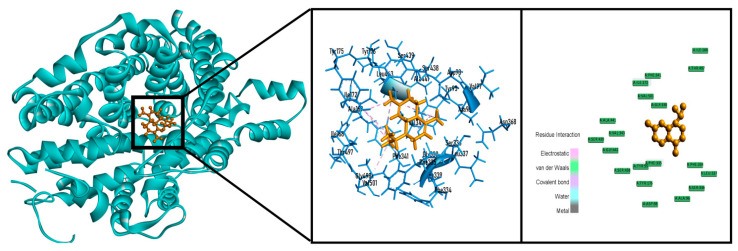
Best docked poses and 2D interactions of alpha-Cadinene with human serotonin receptor (PDB: 5I6X) for antidepressant activity.

In the case of the antidepressant activity assay, the compounds T-cadinol (−6.892 kcal/mol), oplodiol (−6.948 kcal/mol) and α-cadinene (−7.188 kcal/mol) of essential oils in leaves of *H. aromatica* showed the docking score near about while standard drug imipramine hydrochloride has 8.171 kcal/mol docking score (Table 5). α-Cadinene presented a better docking score than other compounds present in leaves of *H. aromatica* against human serotonin receptor by interacting with four hydrophobic bonds (ILE172, VAL501, TYR95 and PHE341) (Figure 9).

From these above outcomes, it can be concluded that the studied phytochemicals of the MEHA may in part be accountable for the antioxidant, thrombolytic, anxiolytic and antidepressant activities through interfaces with these objective receptors. 

**Figure 10:**
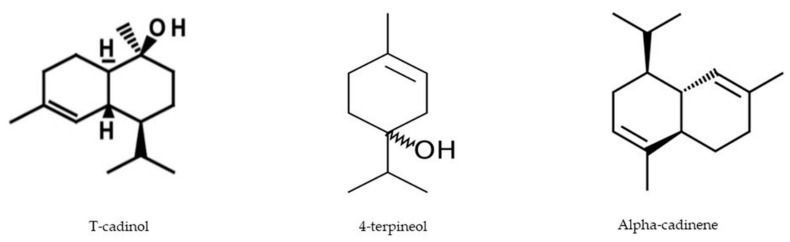
Chemical structures of major compounds identified based on computational study.

There are three major compounds which have been identified based on the computational study showing the best docking scores (Figure 10). It has been observed that T-cadinol showed the best results in the antioxidant as well as thrombolytic assays against xanthine oxidoreductase and tissue plasminogen activator receptor, respectively. The compound 4-terpineol exhibited the best anxiolytic results while α-cadinene displayed the greatest binding affinity for antidepressant activity. Moreover, It has been previously reported that essentials oils have the capability to show thrombolytic [42], anxiolytic [43] and antidepressant [44] activities.

According to their uppermost docking score against xanthine oxidoreductase, human serotonin receptor, potassium channel receptor, tissue plasminogen activator receptor, we have selected 13 phytocompounds in order to find their probable pharmacokinetic factors from drug likeness point of views with their toxicological properties. These sort of categorizations are considered to be a vital phase in the drug development process because it saves time in clinical trials besides expenditures of money. In the current study, SwissADME, an online tool was, used to estimate the pharmacokinetic properties of the 13 selected compounds based on Lipinski’s rule of five and Veber’s rules (Table 6). Oral bioavailability is the vital feature for the development of novel candidate. As described by Lipinski’s rules, an orally introduced drug should have a MW <500 amu, HBA sites <10, HBD sites <5, and, Log P ≤5, whereas Veber et al. recommend that a drug candidate should have the nRB value ≤10 and TPSA value ≤140 Å^2^ [45]. If any compounds violate one of these rules, it cannot be considered a perfect therapeutic agent. Surprisingly, none of the compounds violated these rules in this study. This indicates that all of the compounds have good oral bioavailability and could be considered as possible candidates for the next drug development. 

Furthermore, the admetSAR online tool was used to predict the toxicological properties of the 13 selected compounds. Results of the present study showed that except for α-cadinene and linalyl acetate none of the substances posed a risk of Ames toxicity, carcinogenicity, acute oral toxicity, and weak rat acute toxicity (Table 7). Additionally all of these 13 compounds demonstrated comparatively close human oral bioavailability and human intestinal absorption rate as standard drugs (Table 8). As a result, all 13 phytochemicals could be considered for potential therapeutic candidates with good oral bioavailability though advanced studies are still obligatory.

## 3. Materials and Methods

### 3.1. Reagents, Chemicals and Instruments

Analytical grade chemicals were used in all the experiments. Methanol, ascorbic acid, DPPH (1,1-diphenyl-2-picrylhydrazyl), Folin-Ciocalteau reagent (FCR), sodium carbonate, gallic acid, potassium acetate, aluminium chloride, quercetin were purchased from Sigma Chemicals (St. Louis, MO, USA). Streptokinase, Tween 80, diazepam, imipramine hydrochloride were received from Square Pharmaceuticals Ltd. (Dhaka, Bangladesh). A UV-spectrophotometer (UVmini-1240, Shimadzu, Shimadzu Corporation, Kyoto, Japan) was used for absorbance measurements.

### 3.2. Collection, Identification and Preparation of Extract

Leaves of the *H. aromatica* were collected from Kaptai, Sitapahar, Chittagong, Bangladesh in the month of February 2018. It was identified by Dr. Shaikh Bokhtear Uddin, Professor, Department of Botany, University of Chittagong, Chittagong-4331, Bangladesh. The voucher specimen (accession number: 3286) was deposited in the herbarium center of University of Chittagong. The collected fresh leaves were washed and cut into small pieces and dried under shade for a week. Final dried was carried out in an oven at 40–45 °C for 24 h. A grinder was used to grind the dried leaves into coarse powder. The ground leaves were soaked in adequate amount of methanol for a week at room temperature with vigorous shaking and stirring. The solution was filtered through a cotton-plugged funnel followed by Whatman filter paper number 1. Finally, with the help of rotary evaporator the solvent was evaporated. The viscous mass was kept in a freezer (−20 °C) until further use.

### 3.3. Antioxidant Activity

#### DPPH (1,1-diphenyl-2-picrylydrazyl) Radical Scavenging Activity

DPPH free radical scavenging activity of MEHA was assessed by following the method of Braca et al. [46]. This method has been simplified by Adnan et al. [47]. Briefly, MEHA (100 μL) at various concentrations (50–800 μg/mL) were added to 3 mL of a 0.004% methanol solution of DPPH. After 30 min incubation absorbance was measured at 517 nm and percentage of scavenging activity was determined by using the equation: Percentage (%) of scavenging activity = [(A_0_ − A_1_)/A_0_] × 100. Where, A_0_ is the absorbance of control and A_1_ is the absorbance of extract sample. After preparing the curve, IC_50_ value was calculated using linear regression analysis. The result was obtained in triplicate and expressed as mean ± SEM.

### 3.4. Total Phenolics Content

The total phenolics content of MEHA was determined using Folin-Ciocalteu reagent following the method described by Singleton et al. [48]. Briefly, MEHA (200 μL) was mixed with 400 μL of Folin-Ciocalteu reagent and 1.5 mL of 20% sodium carbonate. The mixture was shaken thoroughly followed by addition of distilled water to make a volume up to 10 mL, and then allowed to stand for 2 h. The absorbance was measured at 765 nm by UV-Spectrophotometer. The concentration of total phenolics content in the extract (10–200 μg/mL) was determined using gallic acid standard curve and results were expressed as mg gallic acid equivalent (GAE) per g of extract (mg GAE/g). The test was analyzed and results were articulated as mean ± SEM.

### 3.5. Total Flavonoids Content

Total flavonoids content was measured following a method described by Kumaran et al. [49] using quercetin as a reference standard. Briefly, 1 mL of MEHA in methanol (10 mg/mL) was mixed with 1 mL aluminum trichloride in methanol (20 mg/mL) and a drop of acetic acid, and then diluted with methanol to 25 mL. The mixture was allowed to stand for 40 min followed by measuring the absorbance at 415 nm. Finally, total flavonoid content was determined using quercetin (12.5–100 μg/mL) standard calibration curve. The values were expressed as mg of quercetin equivalent (QE) per g of extract (mg QE/g). The test was performed for three times and results were expressed as mean ± SEM. 

### 3.6. Thrombolytic Activity

This experiment was done according to the method delineated by Prasad et al. [50]. Briefly, 5 mL sterile distilled water was added in the commercially available lyophilized streptokinase vial (1,500,000 IU) and mixed uniformly for using it as a stock solution. Appropriate dilution was prepared from the stock solution. Next, 5 mL of venous blood was drawn from the healthy volunteers (*n* = 10) without having the history of oral contraceptive or oral anticoagulant therapy as well as having normal physiological parameters (blood pressure, heart rate, BMI and body temperature). The collected blood was distributed (0.5 mL/tube) to each ten previously weighed sterile micro centrifuge tube and incubated at 37 °C for 45 min to form the clot. After clot formation serum was completely removed without dislodged the clot and clot weight was determined from each tube by again measuring the tube weight. Then, 100 μL of MEHA (10 mg/mL) was added to each micro-centrifuge tube containing pre weighed clot. A volume of 100 μL streptokinase as a positive control and 100 μL distilled water as a negative control were separately added to the marked control tubes. For clot lysis observation, all the tubes were then incubated for 90 min at 37 °C. After incubation, released fluid was removed and the tubes were weighed again to observe the difference in weight after clot disruption. The percentage of clot lysis was determined by taking the difference in weight before and after clot lysis.

### 3.7. In Vivo Neuropharmacological Activity

#### 3.7.1. Experimental Animals

Five week-old Swiss albino mice weighing 28–32 g were purchased from animal house of Department of Pharmacy, Jahangirnagar University, Savar, Dhaka, Bangladesh. The mice were acclimatized to the new environment for a week before starting of experiment. During experiment period all animals were maintained in a well-ventilated animal house at 25 ± 2 °C and relative humidity of 50–60%. Standard food pellets and fresh drinking water were supplied throughout the experimental periods. All mice were kept in cage and maintained with natural 12 h light and dark cycle. This animal experiment was designed on the basis of the Ethical Principles and Guidelines guided by the Swiss Academy of Medical Sciences and the Swiss Academy of Sciences. The study protocol was permitted by both the Ethical review committee and the P&D committee of the Department of Pharmacy, International Islamic University Chittagong, Bangladesh under the code Pharm-P&D-61/0818-122.

#### 3.7.2. Acute Toxicity Study

The allocated mice were fasted overnight followed by single oral administration of MEHA (500, 1000 and 2000 mg/kg, body weight). The untreated mice were served as negative control. Mice were checked individually for any physical abnormal behavior and symptoms including allergic syndromes like skin rash, itching, swelling, respiratory complexity and mortality for next 72 h. After that, the mice were euthanized by CO_2_ inhalation and were monitored for respiratory cessation and left in the chamber for 1 min after the respiration has stopped. 

#### 3.7.3. Anxiolytic Activity

##### Elevated Plus Maze Test

The test was accomplished based on the method described by Pello et al. [51]. We concurred the method used by Adnan at al. [52]. Briefly, the apparatus which was elevated 40 cm from the floor containing two open arms (5 cm × 10 cm) and two closed arms (5 cm × 10 cm × 15 cm) that were merged together in a central platform (5 cm × 5 cm) and exposed as plus sign symbol. The mice were distributed randomly into control, standard and treatments groups where each containing 3 mice. Treatment groups mice were orally administered 200 and 400 mg/kg body weight of MEHA, whereas control groups mice received vehicle (1% Tween 80 dissolved in water, 10 mL/kg) orally; and diazepam (1 mg/kg, intraperitoneally) was administered to the standard groups. After 30 min, each mice was kept in the middle of the platform, facing to closed arms and allowed free movement for 5 min. During experiments, open arms entries and total time spent by mice was recorded. 

##### Hole Board Test

The hole board apparatus was used as delineated by Clark et al. [53]. In this experiment, a grid-pattern containing 16 holes of 3 cm diameter in a flat platform with an enclosed space (40 cm × 40 cm × 25 cm) setting up by elevating 25 cm from the floor was used. The instrument contains a hole in the middle of the two connecting frames. The control (1% Tween 80, 10 mL/kg, p.o), standard (diazepam, 1 mg/kg, i.p) and treatment (200 mg/kg and 400 mg/kg, p.o) groups’ mice were allowed to have free movement from middle of the board after 30 min of treatment. Total number of head dipping through the holes were counted for 5 min during the exploration by mice. 

#### 3.7.4. Antidepressant Activity

##### Tail Suspension Test

The tail suspension method was described by Steru at al. [37]. It is a simple and reliable method for screening of antidepressant agents. After 30 min of administration of control (1% Tween 80, 10 mL/kg, p.o) standard (imipramine hydrochloride, 10 mg/kg b.w, i.p) and treatment groups (200 and 400 mg/kg b.w, p.o), the mice were induced in a depression state (immobility), hanging by end of the tail nearly 1 cm from tip of the tail using an adhesive tape. Total immobile time was recorded for last 4 min of a total 6 min (initial adjustment time 2 min) of hanging.

##### Forced Swimming Test

This test was executed as delineated by Porsolt et al. [36]. The experiment was designed to carry out in two session, for example, introductory test was conducted the day before the final experiment for adapting the mice with new environment. A transparent glass tank of 25 cm × 15 cm × 25 cm filled up to 15 cm with water of 25 ± 1 °C temperature was used for swimming. After 30 min of treatment, the treated mice of each groups, for instance, treatments (200 and 400 mg/kg b.w, p.o), standard (imipramine hydrochloride, 10 mg/kg b.w, i.p) and control (1% Tween 80, 10 mL/kg, p.o) was individually placed in the tank for 6 min where initial adjustment time 2 min and the immobility duration were measured for next 4 min.

### 3.8. Protein and Chemical Compounds Studied in This Investigation

From the depository of PDB, 4 proteins were considered for the purpose of this study; xanthine oxidoreductase (PDB: 2CKJ), tissue plasminogen activator receptor (PDB: 1A5H), potassium channel receptor (PDB: 4UUJ), and human serotonin receptor (PDB: 5I6X). The biomolecules studied in this research are included in the essential oils obtained from the leaves of the *H. aromatica.* These were; 1,2-dimethylbenzene (CID: 7237), 4-terpineol (CID: 11230), α-cadinene (CID: 12306048), α-cadinol (CID: 10398656), α-pinene (CID: 6654), bullatantriol (CID: 71430886), camphene (CID: 6616), *cis*-linalool oxide (CID: 6428573), cryptone (CID: 92780), linalyl acetate (CID: 8294), nerol (CID: 643820), oplodiol (CID: 12313756), spatulenol (CID: 92231) and T-cadinol (CID: 160799).

### 3.9. In Silico Studies

#### 3.9.1. Molecular Docking: Preparation of Ligands 

The 2D SDF format of identified compounds was attained from PubChem database of chemical repository (https://pubchem.ncbi.nlm.nih.gov/ (accessed on 1 November 2020)) [54]. LigPrep (bioinformatics tool, part of the Schrödinger -Maestro v 11.1 suite, New York, NY, USA) was used to execute ligand-preparation process [55]. The following factors were kept in consideration during this job: chemical structure was setup as project table, the force field: OPLS3, the target pH: 7.0 ± 2.0 using Epik 2.2.

#### 3.9.2. Molecular Docking: Preparation of Proteins/Enzymes

The receptors proteins were received from the database of Protein Data Bank (PDB) (https://www.rcsb.org/ (accessed on 1 November 2020)). The structures (3D) were taken in PDB format. Pre-processing and minimization step were completed through Protein-Preparation wizard also included in the Schrödinger Maestro v 11.1 suite [55]. The factors that were kept in consideration during this work: the structures optimization pH: 7 and protein minimization done by using OPLS3 force-field. Lastly, the receptor grid selected by using PockDrug which is an online tool for picking the finest binding site.

#### 3.9.3. Molecular Docking: Glide Standard Precision Docking

The docking was executed to assume the conceivable mechanism of the regarding compounds along with the comparison against reference drugs with the associated receptors along with several pharmacological activities. The operation was accomplished by the Ligand-Docking tool of the Schrödinger Maestro software (v 11.1) suite [55]. Afterward, the spreadsheets were collected for additional analysis. Discovery Studio was utilized for better understanding the interaction with 3D imaging. 

#### 3.9.4. Prediction of the Pharmacokinetic Parameters (ADME) 

Several pharmacokinetic properties (absorption, distribution, metabolism, excretion) are of concern during the development process of a therapeutic drug. To describe the numerous biochemical properties the following parameters were explored by SwissADME based on the Lipinski’s and Veber’s Rules [56]: molecular weight (MW), hydrogen bond acceptor (HBA), hydrogen bond donor (HBD), lipophilicity (log P value), number of the rotatable (nRB) and topological polar surface area (TPSA).

#### 3.9.5. Prediction of Toxicological Properties

Determination of toxicological properties is the most crucial in case of the advancement of new therapeutic candidate. A bio-informatics tool named AdmetSAR was castoff to assess the toxicological properties of selective compounds [57]. Various parameters such as rat acute toxicity, acute oral toxicity, Ames toxicity, and carcinogenic properties were kept the focus of these tests.

### 3.10. Statistical Analysis

Data were analysed by using one way ANOVA followed by Dunnett’s multiple comparison tests. GraphPad Prism version 8 software (GraphPad Software Inc., San Diego, CA, USA) was used and the results expressed as mean ± standard error of mean (SEM) where *p* < 0.05 was considered statistically significant.

## 4. Conclusions

The obtained results support the notion that *H. aromatica* possesses medicinal value for the treatment of thrombotic, anxiety and depressive disorders. The phytochemical assessment for antioxidant, phenol and flavonoid contents reinforce the potential of using this plant as a source of anxiolytic and antidepressant treatments. Moreover, the presence of antioxidant and polyphonic compounds make this plant as important choice for treatment of blood clotting ailments. Altogether, these outcomes strongly corroborate the folklore use and popularity of this plant. Furthermore, in silico studies of bioactive compounds have shown promising binding affinities towards different receptors in a molecular docking exploration. The possible pharmacological activities, safety, toxicological properties of the bioactive compounds establish the use of this plant as a promising therapeutic source. Therefore, *H. aromatica* can be considered as a candidate for the development of new drugs. However, comprehensive studies are still needed to isolate and purify novel bioactive leads to reveal the biological activity and mechanism of the observed pharmacological activities.

## Figures and Tables

**Table 1:** Total phenolics and flavonoids content of MEHA.

Sample	Total Phenolics Content (mg GAE/g Extract)	Total Flavonoids Content (mg QE/g Extract)
MEHA	147.716 ± 5.07	66.65 ± 6.208

Each value is represented as mean ± SEM of three independent experiments. GAE, gallic acid equivalent; QE, quercetin equivalent.

**Table 2:** Docking score of the phytochemical compounds of essential oil in leaves of *H. aromatica* against xanthine oxidoreductase (PDB: 2CKJ) for antioxidant activity (Bold indicates lower docking score)**.**

Compounds	Binding Score (kcal/mol)	Glide Emodel (kcal/mol)	Glide Energy (kcal/mol)
1,2-Dimethylbenzene	−4.214	−15.464	−13.480
4-Terpineol	−6.457	−23.043	−16.968
α-Cadinene	-	-	-
α-Pinene	−4.487	−12.354	−11.451
Bullatantriol	−6.260	−34.646	−27.570
Camphene	−4.470	−7.083	−6.289
*cis*-Linalool oxide	−4.825	−30.566	−24.083
Cryptone	−5.259	−24.619	−19.376
Linalyl acetate	−5.116	−34.935	−29.617
Nerol	−4.505	−26.080	−23.519
Oplodiol	−6.182	−33.777	−25.516
Spatulenol	−5.343	−24.144	−19.953
T-Cadinol	**−6.882**	−32.631	−24.531
Ascorbic acid	−6.407	-	-

**Table 3:** Docking score of the phytochemical compounds of essential oil in leaves of *H. aromatica* against tissue plasminogen activator receptor (PDB: 1A5H) for thrombolytic activity (Bold indicates lower docking score).

Compound	Binding Score (kcal/mol)	Glide Emodel (kcal/mol)	Glide Energy (kcal/mol)
1,2-Dimethylbenzene	−4.260	−19.110	−15.447
4-Terpineol	−5.254	−25.836	−20.179
α-Cadinene	−5.720	−34.444	−25.730
α-Pinene	−3.975	−19.073	−15.385
Bullatantriol	−4.350	−31.223	−25.604
Camphene	−3.825	−20.110	−16.344
*cis*-Linalool oxide	−3.794	−27.360	−23.111
Cryptone	−4.537	−25.031	−19.913
Linalyl acetate	−2.781	−24.314	−21.364
Nerol	−2.702	−24.865	−21.837
Oplodiol	−5.075	−32.355	−25.009
Spatulenol	−6.190	−31.911	−25.145
T-Cadinol	**−6.300**	−35.571	−26.388
Streptokinase	−5.102	-	-

**Table 4:** Docking score of the phytochemical compounds of essential oil in leaves of *H. aromatica* with potassium channel receptor (PDB: 4UUJ) for anxiolytic activity (Bold indicates lower docking score).

Compounds	Binding Score (kcal/mol)	Glide Emodel (kcal/mol)	Glide Energy (kcal/mol)
1,2-Dimethylbenzene	−3.658	−11.707	−9.535
4-Terpineol	**−5.143**	−24.454	−20.685
α-Cadinene	−3.691	−12.544	−10.088
α-Pinene	−2.928	−12.540	−10.571
Bullatantriol	−4.098	−23.075	−16.971
Camphene	−2.884	−11.854	−10.084
*cis*-Linalool oxide	−3.512	−25.666	−21.378
Cryptone	−3.442	−18.344	−14.733
Linalyl acetate	−1.155	−16.838	−16.182
Nerol	−2.924	−24.224	−21.507
Oplodiol	-	-	-
Spatulenol	−2.810	−17.092	−14.612
T-Cadinol	−2.457	−6.109	−5.696
Diazepam	3.140	-	-

**Table 5:** Docking score of the phytochemical compounds of essential oil in leaves of *H. aromatica* with human serotonin receptor (PDB: 5I6X) for antidepressant activity (bold indicates lower docking score).

Compounds	Binding Score (kcal/mol)	Glide Emodel (kcal/mol)	Glide Energy (kcal/mol)
1,2-Dimethylbenzene	−6.105	−27.029	−19.783
4-Terpineol	−5.078	−27.507	−20.217
α-Cadinene	**−7.188**	−37.235	−27.179
α-Pinene	−5.426	−21.376	−16.141
Bullatantriol	−6.070	−43.055	−32.486
Camphene	−5.729	−26.921	−19.908
*cis*-Linalool oxide	−5.418	−38.724	−29.151
Cryptone	−5.946	−27.914	−20.359
Linalyl acetate	−3.648	−31.580	−26.453
Nerol	−4.254	−29.861	−24.531
Oplodiol	−6.948	−39.856	−29.137
Spatulenol	−5.796	−32.639	−23.429
T-Cadinol	−6.892	−40.513	−28.388
Imipramine hydrochloride	8.171	-	-

**Table 6:** ADME profiling of the phytochemical compounds of essential oil in leaves of *H. aromatica.*

Compounds	Lipinski Rules				Lipinski’s Violations ≤1	Veber Rules	
	MW (g/mol) <500	HBA <10	HBD <5	Log P ≤5		nRB ≤10	TPSA ≤140
1,2-Dimethylbenzene	106.17	0	0	2.03	No	0	0.00 Å^2^
4-Terpineol	154.25	1	1	2.51	No	1	20.23 Å^2^
α-Cadinene	204.35	0	0	3.38	No	1	0.00 Å^2^
α-Cadinol	222.37	1	1	3.15	No	1	20.23 Å^2^
α-Pinene	136.23	0	0	2.63	No	0	0.00 Å^2^
Bullatantriol	256.38	3	3	2.60	No	2	60.69 Å^2^
Camphene	136.23	0	0	2.58	No	0	0.00 Å^2^
*cis*-Linalool oxide	170.25	2	1	2.45	No	2	29.46 Å^2^
Cryptone	138.21	1	0	2.05	No	1	17.07 Å^2^
Linalyl acetate	196.29	2	0	3.08	No	6	26.30 Å^2^
Nerol	154.25	1	1	2.75	No	4	20.23 Å^2^
Oplodiol	238.37	2	2	2.98	No	1	40.46 Å^2^
Spatulenol	220.35	1	1	2.88	No	0	20.23 Å^2^
T-Cadinol	222.37	1	1	3.15	No	1	20.23 Å^2^

MW, Molecular weight; HBA, Hydrogen bond acceptor; HBD, Hydrogen bond donor; Log P, Lipophilicity; nRB, Number of rotable bond; TPSA, Topological polar surface area.

**Table 7:** Toxicological properties of the phytochemical compounds of essential oil in leaves of *H. aromatica.*

Compounds		Parameters			
		Ames Toxicity	Carcinogens	Acute Oral Toxicity	Rat Acute Toxicity (LD_50_, mol/kg)
1,2-Dimethylbenzene		NAT	NC	III	1.5513
4-Terpineol		NAT	NC	III	2.0424
α-Cadinene		AT	NC	III	1.4298
α-Cadinol		NAT	NC	III	2.2009
α-Pinene		NAT	NC	III	1.5348
Bullatantriol		NAT	NC	III	2.8543
Camphene		NAT	NC	III	1.4664
*cis*-Linalool oxide		NAT	NC	III	2.1384
Cryptone		NAT	NC	III	1.8337
Linalyl acetate		NAT	C	IV	1.4627
Nerol		NAT	NC	III	1.6146
Oplodiol		NAT	NC	III	2.9423
Spatulenol		NAT	NC	III	2.5610
T-Cadinol		NAT	NC	III	2.2009
Standards	Ascorbic acid	NAT	NC	IV	1.3059
	Streptokinase	NAT	NC	II	2.2785
	Diazepam	NAT	NC	II	2.5946
	Imipramine hydrochloride	NAT	NC	III	2.8931

NAT, Non-Ames toxic; AT, Ames toxic; C, Carcinogenic; NC, Non-carcinogenic. Category-I means (LD_50_ ≤50 mg/kg) and Category-III (500 mg/kg LD_50_ 5000 mg/kg).

**Table 8:** Additional pharmacokinetic profiling (absorption and oral bioavailability) of the phytochemical compounds of essential oil in leaves of *H. aromatica.*

Compounds		Human Intestinal Absorption	Human Oral Bioavailability
1,2-Dimethylbenzene		0.9801	0.9143
4-Terpineol		0.9842	0.6286
α-Cadinene		0.9778	0.7429
α-Cadinol		0.9892	0.5143
α-Pinene		0.9677	0.8286
Bullatantriol		0.9841	0.6571
Camphene		0.9677	0.6286
*cis*-Linalool oxide		0.9626	0.5857
Cryptone		0.9905	0.6857
Linalyl acetate		0.9703	0.6143
Nerol		0.9681	0.5429
Oplodiol		0.9896	0.5857
Spatulenol		0.9906	0.7330
T-Cadinol		0.9892	0.5143
Standards	Ascorbic acid	0.8150	0.5857
	Streptokinase	0.4612	0.5857
	Diazepam	0.9948	0.9286
	Imipramine hydrochloride	0.9920	0.7286

## Data Availability

The data presented in this study are available on the request from the corresponding author.

## Sample Availability

Samples of the compounds are not available from the authors.

## Abbreviations

PDB	Protein data bank
GABA	Gamma amino butyric acid
p.o	per oral
i.p	intraperitoneal
ADME/T	Absorption, distribution, metabolism, excretion and toxicity
BMI	Basal metabolic rate
ANOVA	Analysis of variance
SPSS	Statistical package for social science
SEM	Standard error of mean
